# QuTILs: Open-Source Image-Based Infiltrating Immune Cell Detection for Research Application

**DOI:** 10.21203/rs.3.rs-9535059/v1

**Published:** 2026-05-14

**Authors:** Mark Vater, Roberto Salgado, Elijah Blige, Heather Lefebvre, Ava Strahan, Margaret Gatti-Mays, Hope Rugo, Karla Ballman, Mark Watson, Yujia Wen, Roberto Leon-Ferre, Khalid Niazi, W. Fraser Symmans, Lisa Carey, Khalid AbdulJabbar, Zaibo Li, Daniel G Stover

**Affiliations:** The Ohio State University Wexner Medical Center; Gasthuiszusters van Antwerpen Hospital (GZA) and Ziekenhuis Netwerk Antwerpen Hospital (ZNA); The Ohio State University Wexner Medical Center; The Ohio State University Wexner Medical Center; The Ohio State University Wexner Medical Center; The Ohio State University Wexner Medical Center; University of California, San Francisco; Mayo Clinic; Washington University Medical Center; University of Chicago; Mayo Clinic; The Ohio State University Wexner Medical Center; The University of Texas MD Anderson Cancer Center; UNC Lineberger Comprehensive Cancer Center; Case45; Stefanie Spielman Comprehensive Breast Center, The Ohio State University Comprehensive Cancer Center; The Ohio State University Wexner Medical Center

**Keywords:** Breast cancer, digital pathology, tumor infiltrating lymphocytes

## Abstract

Stromal tumor infiltrating lymphocytes (sTILs), quantified via hematoxylin and eosin (H&E) tumor slides, are associated with improved response to chemotherapy and better overall survival (OS) in triple-negative breast cancer (TNBC). Digitization of H&E slides offers the opportunity for image-based computational approaches to enumerate sTILs. We describe QuTILs, a research-based TIL enumeration approach using a multilayer perceptron-based framework trained on open-source H&E images using the QuPath software and executed on a standard computer. QuTILs was applied to H&E images from two phase III TNBC clinical trials, CALGB 40502 and 40603 (total n = 462 patients). In Cox proportional hazards models, QuTILs showed significant univariate association in CALGB 40502, with higher TIL% associated with reduced hazard (HR: 0.75, 95% CI: 0.63–0.91), which remained significant in multivariable models and validation CALGB 40603 TNBC clinical trial. In summary, QuTILs provides a computationally efficient, open-source workflow for sTIL identification from digital H&E images for research application.

## INTRODUCTION

The breast cancer (BC) tumor-immune microenvironment (TIME) represents a complex ecosystem of diverse cell types – cancer cells, stroma, and immune cells, including tumor infiltrating lymphocytes (TILs) (1–3). Pathologists’ quantification of stromal TILs (sTILs) via hematoxylin and eosin (H&E) tumor slides (following International TILs/Immuno-Oncology Working Group (IOWG) guidelines) is an effective surrogate for host antitumor immunity (4–6). There are established associations between sTILs and relevant clinical outcomes in BC: sTILs are prognostic and associated with response to neoadjuvant chemotherapy in primary triple negative breast cancer (TNBC) and HER2 + BC and prognostic in residual tissue after neoadjuvant chemotherapy in TNBC (7–10).

Among approaches to interrogate the TIME, sTILs via H&E offer an accessible and consistent vehicle to characterize the TIME. While sTILs are not yet routinely used for clinical care, sTILs are comparable to other immune biomarkers in BC, such as PD-L1 immunohistochemistry (IHC) and RNA-sequencing (RNA-seq)-based metrics (11–14). Further, sTILs are a low-cost approach, particularly relative to bulk tumor or single-cell RNA-seq-based immune metrics. While sequencing-based approaches have revolutionized our understanding of specific immune phenotypes, it is unclear how sTILs and RNA-seq-based approaches may overlap or complement one another, particularly as predictive or prognostic biomarkers.

Clinical application of sTIL estimations has historically relied upon a pathologist manually and directly annotating cells from pathology slides (4). Digitization of H&E slides has gained acceptance for use in diagnostic pathology and research, opening the door for image-based computational approaches (15–17). In recent years, with the advent of convolutional deep neural networks (CNNs), there have been major breakthroughs in implementing accurate automated estimations of image-based TILs (18–20). Bai et al. introduced CNN11, a multilayer perceptron (MLP)-based classifier utilizing the open-source QuPath software, which was highly predictive for prognosis in medical BC datasets (21, 22). However, CNN11 is not functional with the current version of QuPath at time of writing, leaving an important gap in open-source TIL enumeration research. AbdulJabbar et al. demonstrated a CNN pipeline trained across 26,000 cells with high predictive accuracy for immune, tumor, stromal, and other cells, yet requires a high-performance computing environment with graphical processing unit (GPU) capacity (23). Further, while advanced computational methods have demonstrated initial success, these models typically cannot be deployed on standard computing devices (24, 25). This reinforces the need for classifiers within the constraints of basic computer environments that researchers can readily apply.

The aims of this investigation were 1) to create, implement, and benchmark an open-source automated algorithmic estimation of TILs from histological images for research application, termed ‘QuTILs’; 2) to assess the relationship between TIL patterns generated by QuTILs with clinical outcomes across two BC cohorts; 3) to evaluate the association between image-based TIL levels and RNA-seq-based immune metrics.

## MATERIALS AND METHODS

### Clinical Cohorts

Publicly-available digital pathology data (digitized H&E images) from The Cancer Genome Atlas were used as training data. Additionally, two phase III clinical trials with TNBC participants were identified for inclusion in this study based on availability of primary BC digital H&E slides with paired RNA-seq data: Cancer and Leukemia Group B (CALGB) 40502 (NCT00785291), and CALGB 40603 (NCT00861705) (26–28). Protocol-specific written informed consent was obtained from participants, and the protocol was approved by the National Cancer Institute’s (NCI’s) Institutional Review Board (IRB). The informed consent document complies with federal and institutional guidelines, including the Declaration of Helsinki, for collection and use of data and samples. Analyses of deidentified pathology image data and RNA-seq was approved through the NCI Navigator approvals CSC0086 and CSC0087, Alliance for Clinical Trials in Oncology approval A152214, and through The Ohio State University IRB #2021C0216. Clinical data for each clinical trial was obtained via National Clinical Trials Network (NCTN) Data Archive (Requests 3021 and 3109). CALGB 40502 was a randomized trial assessing 3 lines of chemotherapy treatment in patients with metastatic or locally recurrent BC (26, 29). CALGB 40603 examined the role of combination anthracycline or taxane neoadjuvant therapies for long-term survival in TNBC (27, 28). CALGB is now part of the Alliance for Clinical Trials in Oncology. The number of enrolled cases and available data for each cohort are shown in **Supplementary Table S1**. The number of images and RNA-seq data were limited to those that could be matched to available survival data. As part of this study, only primary breast tumor H&E images were analyzed; analysis of metastatic biopsies (collected in CALGB 40502) presented unique challenges and will be part of future analyses.

### QuTILs Model Development

To maximize total training data for the model, open-source TIL annotations were acquired from 2 sources using The Cancer Genome Atlas Program (TCGA) H&E data: PanopTILs (includes cell-level TIL annotations, both manually labelled and algorithmically expanded) and TIL hotspot density maps from Saltz et al. (30–32). PanopTILs is an online repository of crowd-sourced pathologist annotations of tumor, stromal, immune, and other cell types as well as tissue regions in TCGA BC slides. This dataset includes coordinate regions on TCGA slides representing individual TIL nuclei annotated directly by pathologists, as well as those that were detected by a machine learning algorithm trained on ground truth data. Specifically, 107 digital H&E slides from TNBC samples with bootstrapped cell labels (training) and 20 TNBC digital H&E slides with pathologist-evaluated ground truth cell labels (validation) were utilized from the PanopTILs dataset, with an additional 33 TNBC digital H&E slides containing TIL cluster hotspots from the Saltz et al. (2018) dataset. The QuTILs model is a MLP model including shape, optical density, and texture features at the individual cell and community levels. More specifically, each cell was classified based on features including shape, size, intensity values, and texture features of the detected nucleus and cell, as well as the average values of those features in small neighborhoods around each estimated cell area.

### QuTILs Workflow

**Figure 1:**
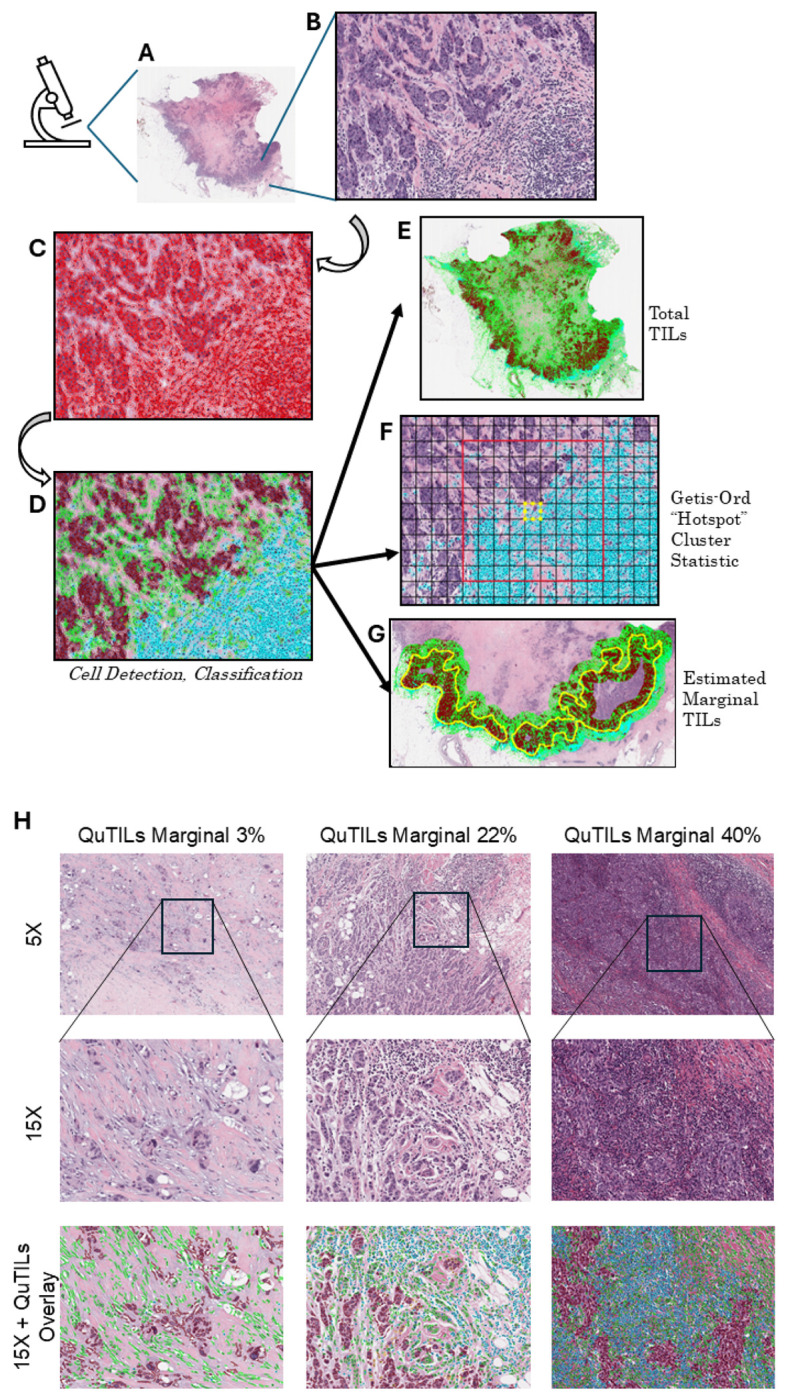
QuTILs Workflow. Hematoxylin and eosin (H&E) tumor slides are first digitized for processing (**A-B),** watershed cell detection is applied to stroma, tumor, and immune classified tissue objects to generate cell objects (**C**) then the QuTILs multi-layer perceptron (MLP)-based cell classifier was applied to detected cell objects, resulting in tumor, stromal, lymphocyte and ‘other’ cells (**D**). The largest contiguous tumor tissue annotations were retained for invasive margin assessment simulation – the borders of these annotations were dilated inward and outward by 500μm, resulting in a restricted region for TIL counting (**E**). All classified TILs were also assessed as to their Euclidean distance from the nearest tumor cell in tumor tissue regions (disregarding isolated or small patches of tumor cells; **F**). and the total tissue region sectioned into smaller tiles of 50x50μm, each tile assessed for underlying TIL count for comparison against the global TIL mean (**G**).

This analysis made use of the popular open-source digital pathology software suite QuPath (22). This software allows users to conduct a variety of routine digital pathology tasks, including tissue and cellular identification, segmentation, and classification. QuPath makes use of OpenCV libraries for classification including random decision trees and MLP analysis (33). All analyses were conducted in QuPath version 0.6.0. QuTILs uses QuPath functions to first divide wholes slide image tissue regions into tiles (Fig. 1A–B) which are then filled with cell objects detected by watershed method and classified as TILs, tumor, stroma, or ‘other’ cells by individual and neighborhood measurements (Fig. 1C–D). Once cells are detected and classified, this allows for extraction of measurements including total whole-slide level TILs, estimated invasive marginal TILs, and cluster ‘hotspot’ TILs percentages (Fig. 1E–G). Estimated invasive margin was defined by first identifying clusters of ≥ 100 tumor cells per 300 μm radius in whole-slide images and merged to form the boundary of estimated invasive margin. In core biopsy images, given variable tissue size, the tumor cell density and patch radius was increasingly lowered until at least five tumor patches were identified if none were generated from the 100 cell/300 μm method. Tumor patches were then dilated outward and eroded inward by 500 μm each to simulate manual invasive margin estimation, producing cell class scores under each generated area.

### Clinical Analysis

To evaluate the possible association of QuTILs measures and survival outcomes, estimates were compared both in univariable and multivariable survival models using available survival data: overall survival (OS) in CALGB 40502 and event-free survival (EFS) in CALGB 40603. Median follow-up time for surviving patients was 25 months (CALGB 40502; date of data lock June 4, 2013) and 7.9 years (CALGB 40603; date of data lock April 2, 2020) (26, 27, 29). To compare the utility of each method of estimating TILs, all TILs metrics were assessed across the three cohorts in a univariate manner via Kaplan-Meier (K-M) curves. For Cox proportional hazards modeling, selected TILs estimates were combined with available survival-relevant medical patient data. For a full description of the methods used, please refer to **Supplement A: Detailed Methods**.

## RESULTS

### QuTILs Model Performance and Benchmarking

QuTILs was developed to serve as a highly accessible benchmarked tool for TIL quantification using a current version of QuPath (ver 0.6.0). Specifically, a main goal was to develop an sTIL approach that could be run on a standard computer. Using a standard-issue laptop computer with an Intel i7 10-core processor and 32 GB DDR5 RAM, the QuTILs workflow processed all sTIL metrics over an average time of 6.7 minutes for core biopsy images and 8.4 minutes for whole-slide images. The QuTILs classifier and workflows are available at: https://github.com/mfvater/qutils. In sum, the QuTILs approach provides a scalable assessment for research TIL determination across the spectrum of TIL infiltration, from low (marginal TILs = 3%), medium (marginal TILs = 22%), and high (marginal TILs = 40%) (Fig. 1H). See **Supplement A: Detailed Methods**.

QuTILs represents a conservatively accurate model for distinguishing TILs from other cell classifications in H&E images, achieving an overall accuracy of 88.1% in a holdout validation dataset, with high specificity of 94.7% and sensitivity of 76.4%, indicating strong likelihood for identification of TILs with high confidence, a primary goal in algorithm development (**Supp** Fig. 1A-B). We benchmarked QuTILs alongside an existing, yet deprecated QuPath-based TIL identifier (CNN11) introduced by Bai et al. in 2021 (**Supp** Fig. 1C-D) (21). The CNN11 algorithm is only available for implementation using outdated QuPath version 0.1.2 with no update available for current QuPath version 0.6.0; thus, comparisons represent implementation on different QuPath versions (21). Additionally, as QuTILs is presented in the form of a generic .json file, that will interface seamlessly with future versions of QuPath. QuTILs achieved an overall higher accuracy (88.11% vs. 83.32%) and F1 score (82.22% vs. 77.61%). QuTILs demonstrated a much higher specificity rate (94.71% vs. 84.76%) at the expense of modestly lower sensitivity (76.36% vs. 80.74%), indicating that QuTILs output is more likely to identify true TILs, while CNN11 captures a greater number of TILs at the expense of accuracy. Overall, QuTILs demonstrates better accuracy, specificity, positive predictive value, and F1 measure, while also utilizing the current QuPath version.

### QuTILs Estimates by Cohort and Demographic Variables

To assess QuTILs across cohorts by available demographic variables, results were assessed between cohort averages using Student’s t-tests for both total estimated % and marginal estimated % TILs (**Supp** Table 1; **Supp** Fig. 2). A significant difference was observed between estimated TILs levels in CALGB 40502 and CALGB 40603, with mean total TILs % higher in CALGB 40502 than CALGB 40602 (*t* = 2.37, *P* = 0.02) as was marginal % TILs (*t* = 2.42, *P* = 0.02), potentially due to larger available tissue area by surgical sections in CALGB 40502 relative to core biopsy samples only in CALGB 40603 (**Supp** Fig. 2A). Regarding estimated TILs levels by available tumor grade data in CALGB 40603, grade 3 tumors exhibited significantly higher total TILs % (*t* = 3.56, *P* < 0.01) and marginal TILs % (*t =* 4.07, *P* < 0.01) than grade I or II cancers, as anticipated (**Supp** Fig. 2B). There were no significant differences between race and age categories by total or marginal % TILs estimates within cohorts (**Supp** Fig. 2C-2F).

#### High versus Low QuTILs and Clinical Outcomes in Testing and Validation Phase III TNBC Clinical Trials

**Figure 2:**
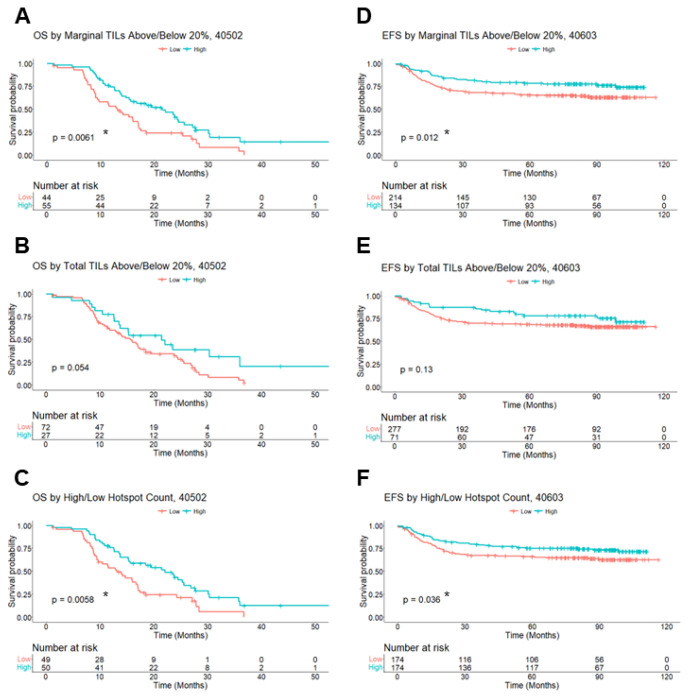
Kaplan-Meier (K-M) Survival Analysis. **A-E**) K-M curves with median cutoff (high/low) for TIL metrics by QuTILs were assessed in the TNBC subset of CALGB 40502 (**A-C**) and CALGB 40603 (**D-F**) – simulated invasive marginal % count (**A, D**), total % count (**B, E**), hotspot density (**C, F**).

The primary clinical endpoint was association of high versus low QuTILs metrics (each stratified by 20% cutoff, as used by previous investigations (28), which approximated previous high/low splits; high TILs vs. low TILs) with survival endpoints across cohorts. Pathologist TILs have demonstrated a significant association with survival and/or treatment response (such that favorable outcomes follow higher TILs) previously (12, 29, 34–36). As testing and validation in two separate BC phase III TNBC clinical trials with, we evaluated the associations in CALGB 40502 and CALGB 40603. In CALGB 40502, as would be expected, higher TILs were associated with significantly better OS by marginal TILs % (median survival: 17.6 vs. 13.0 months, log-rank *P* = 0.0061; Fig. 2A) and by above versus below median ‘hotspot’ number (18.7 vs. 12.8 months, *P* = 0.0058; Fig. 2C), with a non-significant association for total TILs % (Chi-square: 3.7, log-rank *P* = 0.054 Fig. 2B). The CALGB 40603 (TNBC) cohort similarly demonstrated that higher QuTILs metrics were associated with improved EFS, with a significant association for marginal TIL% (median survival: 82.6 vs. 78.8 months, log-rank *P* = 0.012; Fig. 2D) and ‘hotspot’ number (median survival: 81.8 vs. 78.7 months, log-rank *P* = 0.036; Fig. 2F), with a similar non-significant association with total TIL% (Chi-square: 3.0, *P* = 0.13, Fig. 2E).

### Univariable/Multivariable Models with QuTILs Metrics and Clinical Outcomes in Phase 3 Clinical Trials

To assess QuTILs metrics as a continuous variable, univariable and multivariable Cox proportional hazards models were built for the discovery cohort and validation phase III clinical trial (Table 1). Univariable Cox models assessing each predictor in a standalone model, with total TILs %, marginal TILs %, and cluster ‘hotspot’ proportion of total tissue area as QuTILs metrics were constructed, along with age, race, and treatment arm – each implicated as mediators of outcome in these populations. For multivariable models, estimated marginal % TILs were incorporated as the best-performing automated estimate of TILs. Generally, both univariable and multivariable Cox models reflected trends shown in K-M analysis.

The testing CALGB 40502 TNBC cohort showed significant univariate association for total TILs % with increasing decile (higher total TIL%) associated with reduced hazard (hazard ratio [HR]: 0.717, 95% CI: 0.560–0.917; *P* = 0.008) (Table 1). Marginal TIL% also demonstrated increasing decile associated with reduced hazard in univariable (HR: 0.754, 95% CI: 0.626–0.909; *P* = 0.003; Table 1) and multivariable analyses (HR: 0.746, 95% CI: 0.610–0.913; *P* = 0.037). Further, natural log-transformed number of significant ‘hotspots’ per total tissue area was additionally significantly associated with survival in univariable modeling (HR: 0.754, 95% CI: 0.543–0.933; *P* = 0.014). In CALGB 40603, similar to the CALGB 40502 TNBC cohort, marginal TILs % was significant in univariable (HR: 0.789, 95% CI: 0.675–0.922) models, as was total TILs % (HR: 0.796, 95% CI: 0.639–0.994) and log-transformed ‘hotspots’ (HR: 0.850, 95% CI: 0.733–0.985). Further, decile marginal TILs % was similarly associated significantly with survival in the multivariable model (HR: 0.802, 95% CI: 0.685–0.940) (Table 1).

### Association of QuTILs Metrics and RNA-seq Immune Features with Clinical Outcomes

**Figure 3:**
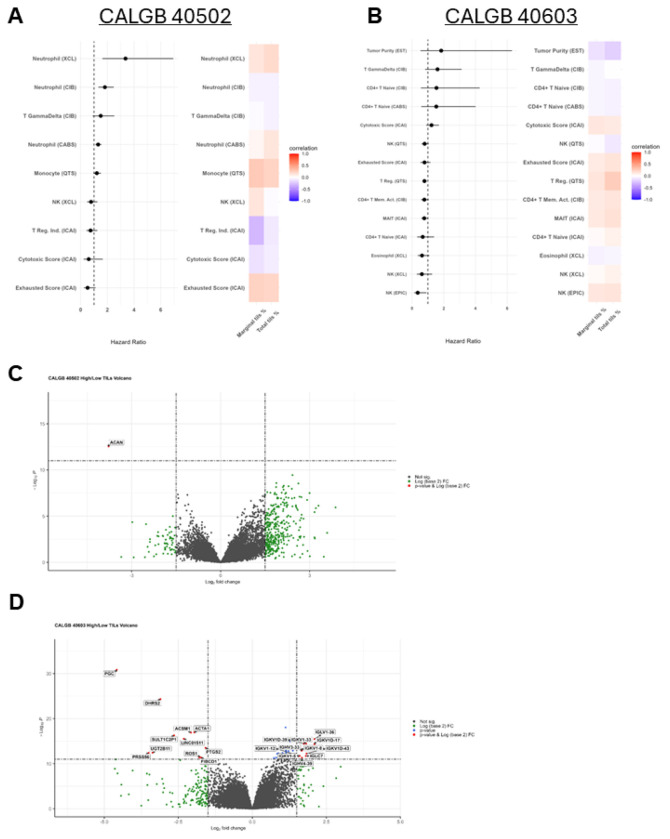
Association of RNAseq Immune Deconvolution Subtypes with QuTILs and Patient Outcome. Cox’s proportional hazards model of immune subtypes across deconvolution methods with overall survival in CALGB 40502 (A) and CALGB 40603 (B), with those subtypes demonstrating hazard ratio either <0.8 or >1.2 included (all metrics are presented in Supplementary Figure 3). Correlation to QuTILs marginal (left) and total (right) TIL estimates are indicated in the adjacent heatmap for each immune subtype.

Currently, TIL enumeration and RNA-seq-based immune deconvolution are two of the most popular research-based approaches to interrogate the TIME in BC samples. Using Cox’s proportional hazards model, we first evaluated the association of immune subtypes across deconvolution methods with survival in the discovery cohort (Fig. 3A–B; **Supp** Fig. 3**)**. Of the 170 categories of immune phenotype and method combinations returned by TIMER3.0, only 17 were significantly associated with survival in CALGB 40502 in the univariable context, and only 10 with survival outcome in CALGB 40603 (**Supp** Fig. 3A-B, respectively). However, no deconvoluted immune estimate remained significantly associated following multiple testing adjustment by either Bonferroni correction or Benjamini-Hochberg method. Since deconvolution approaches may not be optimal, we evaluated the association of single genes with QuTILs based on 20% cutoff for marginal-estimated QuTILs percent (Fig. 3C–D). In CALGB 40502, only one gene, *ACAN*, was found to be significantly attenuated in the QuTILs high group relative to low, with no genes significantly enriched (Fig. 3C). For CALGB 40603, several genes were found to have significantly higher and lower expression levels in the QuTILs high group. Specifically, of note, 11 genes across the *IGL, IGH*, and *IGKV* families were enriched in the high TILs group, all of which are B-cell related genes (Fig. 3D).

To address the important question of whether image-based TIL determinations or RNA-seq-based immune metrics (or both together) are more strongly associated with clinical outcomes, Cox models for the best performing RNA deconvolution (those that were in the top 15 highest and lowest univariable HR by Cox model overlapping across both cohorts) with sTILs were evaluated. In multivariable modeling, QuTILs estimated marginal % TILs by decile was significantly associated with overall survival in the CALGB 40502 cohort and event-free survival in the CALGB 40603 cohort after adjusting for various deconvoluted immune scores (Table 2). While no deconvolution covariates were also significant in this feature set in CALGB 40603, for CALGB 40502 models, estimated monocyte level by quanTIseq method (QTS) was significant alongside marginal TILs %, both in the full rank model and a reduced model with only those two predictors (Table 1). However, these results broadly suggest that RNA-based deconvolution estimates may not supplement the prognostic power of image-based TIL estimates in general, as only one deconvolution-based covariate significantly explained model variance out of 170 total tested deconvolution estimations.

### Pre-treatment and Post-treatment Comparison

CALGB 40603 had both pre-neoadjuvant and post-neoadjuvant therapy H&E images, offering an opportunity for an exploratory analysis of changes in computational image-based QuTILs metrics pre-/post-neoadjuvant therapy. Overall, there was a significant decrease in QuTILs estimated marginal TIL% in CALGB 40603 for all pairs (*n* = 80 pairs, pre-median = 14.46%, post-median = 10.22%; Wilcoxon signed rank *W* = 943, *P* = 0.008; **Supp Fig. 4A-B**) and among only those cases that exhibited a > 5% increase or decrease in percent marginal TILs (*n* = 50 pairs, pre-median = 11.44%, post-median = 8.82%; *W* = 2171, *P* = 0.008; **Supp Fig. 4C-D**). There were several cases that showed an increase in TILs following therapy, many of which had initial TIL estimates up to 20% (**Supp Fig. 4A, 4C**). One acknowledged limitation is the heterogeneity of tissue source (whole section image versus core biopsy) between pre-treatment and post-treatment samples. This is the first analysis of pre-/post-neoadjuvant therapy TILs in a large phase III clinical trial.

## DISCUSSION

Tissue-based TIL estimations have demonstrated significant association with outcome across many studies in BCs, specifically TNBC and HER2 + subtypes (21, 23, 37–40). Until recently, accurate representations of TILs from microscopy images have relied upon pathologists’ manual determination. However, advancements in the fields of computer vision and deep learning have led to automated and digitized approaches for estimation of image-based TILs. Many investigators have successfully incorporated automated image-based TIL estimates into their workflow for evaluation of BC cases, though the vast majority of these are evaluated in medical or aggregate datasets (37–39). In this investigation we demonstrated the utility of a QuPath-based TIL classifier trained on open-source data and evaluated in phase III TNBC clinical trials, specifically with a computationally efficient approach that can be implemented on a standard computer. This investigation offers an open source, readily accessible resource for researchers of diverse computational backgrounds to determine estimated TILs from digital pathology images.

Invasive margin estimates provided by QuTILs were prognostic for TNBC cases as high versus low in simple K-M models and as a continuous variable in univariable and multivariable models across two phase III clinical trials. This is supported by existing results on studies assessing the role of TILs in predicting BC survival (38, 40–43). Further, the application of QuTILs on both pre- and post-neoadjuvant therapy samples revealed that post-treatment QuTIL levels were not informative as a biomarker for survival analysis, as opposed to pre-treatment levels suggesting that application of this straightforward method may provide unique insights.

As computerized approaches have emerged, various strategies for estimating TILs have been proposed. Previous research has suggested that assessment of TILs across a whole slide of tissue may be sufficient for predicting prognosis, or assessment of clusters or “hotspots,” as opposed to estimating within the invasive margin specifically (21, 31, 37, 43). An increasing number of investigations have focused on the potential spatial role of TILs and distance-based metrics for predicting disease severity in BC (37, 44, 45). While many of these metrics have been found to significantly associate with prognosis or treatment response, there is little evidence that any one estimation type outperforms another in terms of association strength or consistency. Relative to CNN11, the most prevalent public release of a QuPath-based TIL classifier, QuTILs presents a desirable alternative. First, the CNN11 implementation is only compatible with a largely outdated version of QuPath, making it more cumbersome than QuTILs. Additionally, QuTILs may present a more conservative approach with a slightly lower sensitivity but much higher specificity for accurate segmentation of TIL cells. The authors of CNN11 also showed examples of TIL misclassification, which were not seen in the implementation of QuTILs in these data (21). Overall, this work has established the usefulness of QuTILs for prognostic assessment of TNBC in clinical trials supporting the utility of QuTILs as a research tool.

Quantification of sTILs via H&E tumor slides (either via pathologists or computationally) offers a widely available, low-cost method to define a simple yet effective surrogate for host antitumor immunity (46), particularly relative to more costly genomic approaches, such as bulk or single-cell RNA-seq. Several methods have been produced to estimate the abundance of specific immune phenotypes from RNA-seq data, including CIBERSORT, EPIC, quanTIseq, MuSiC, TIMER, xCell, and BayesPrism (47–49, 52–60). These methods are purely *in silico* in nature, providing regression-based and/or signal gene estimates of immune cells (50, 51). Many such methods are validated against single-cell quantifications and large datasets; while there is an obvious need for the quantification of specific immune cells without the expense and time constraints of single-cell methods, the application of these estimates in clinical modeling remains uncertain (50, 51). Estimates from RNA deconvolution methods have shown significance in specific cohorts and investigations in the past but may not perform consistently across datasets (61, 62). Prior work from S. Badve and colleagues demonstrated that pathologist enumerated sTILs and RNAseq-based CIBERSORT are largely discordant in TCGA (61). We interpret these results to suggest that TILs and RNA-based deconvolution estimates are complementary.

There are several limitations of our study. Training data were drawn from TCGA slide images, which are exclusively whole-slide tissue samples post-surgery. This allowed capture of a wide range of staining variation, for instance, but many of the slides that the QuTILs method was evaluated on were core biopsy samples, which may be impacted by quality control or standardization. Our approach to capturing and representing the invasive margin is an initial approach and can be further refined. QuPath has recently integrated a deeper learning library through Java, and retraining a deeper QuTILs model may increase the accuracy of estimates. Additionally, incorporating a wider range of RNA deconvolution methods may assist with the informativeness of the combination image-and-RNA strategy.

In conclusion, QuTILs provides a computationally efficient open-source workflow for sTIL identification from digital H&E images that can be deployed on a standard workstation or laptop using the current version of QuPath. QuTILs enumeration of TILs demonstrates significant association with outcomes among patients with TNBC in large phase III TNBC clinical trials.

## Supplementary Material

This is a list of supplementary files associated with this preprint. Click to download.

- SUPPLEMENTARYTABLESFIGURESupdated.docx
- MethodsSuppupdated.docx

## Figures and Tables

**Table 1:** Cox’s Hazards Models – QuTILs and Cohort Features. *Univariable Multivariable*

	HR	95% CI	*p*	HR	95% CI	*p*
**CALGB 40502**						
**Total TILs % (decile)**	**0.717**	**0.560–0.917**	**0.008**			
**Significant Cluster ‘Hotspot’ proportion (nat. log)**	**0.711**	**0.543–0.933**	**0.014**			
**Marginal TILs % (decile)**	**0.754**	**0.626–0.909**	**0.003**	**0.746**	**0.610–0.913**	**0.004**
Age (20–49 vs. 50–69)	1.363	0.802–2.317	0.253	1.079	0.608–1.916	0.794
Age (20–49 vs. >69)	1.344	0.533–3.392	0.531	1.258	0.498–3.178	0.628
Race (White vs. Non-white)	0.849	0.480–1.503	0.575	0.848	0.465–1.545	0.589
Treatment arm (bev.+pac. vs. nab-pac.)	0.972	0.558–1.695	0.921	0.861	0.488–1.520	0.606
Treatment arm (bev.+pac. vs. ixa.)	1.196	0.681–2.102	0.534	1.104	0.615–1.981	0.741
**CALGB 40603**						
**Total TILs % (decile)**	**0.796**	**0.639–0.994**	**0.044**			
**Significant Cluster ‘Hotspot’ proportion (nat. log)**	**0.850**	**0.733–0.985**	**0.031**			
**Marginal TILs % (decile)**	**0.789**	**0.675-0,992**	**0,003**	**0.802**	**0.685–0.940**	**0.006**
Age (20–49 vs. 50–69)	1.440	0.880–2.356	0.146	1.392	0.849–2.284	0.190
Age (20–49 vs. >69)	1.478	0.807–2.707	0.205	1.282	0.687–2.389	0.435
Race (White vs. Non-white)	1.141	0.745–1.747	0.545	1.073	0.695–1.658	0.751
Treatment arm (pac.+dox. vs. +bev.)	1.152	0.669–1.985	0.601	1.118	0.647–1.935	0.689
Treatment arm (pac.+dox. vs. +car.)	1.216	0.718–2.060	0.466	1.126	0.662–1.915	0.661
**CALGB 40502**						
**Total TILs % (decile)**	**0.717**	**0.560–0.917**	**0.008**			
Treatment arm (pac.+dox. vs. +bev. +car.)	0.963	0.550–1.686	0.895	0.983	0.560–1.724	0.952
**pCR (non-pCR vs. pCR)**	**0.273**	**0.174–0.428**	**<0.001**			

**Abbreviations**: bev – bevacizumab; pac – paclitaxel; nab-pac – nab-paclitaxel; ixa – ixabepilone; dox – doxorubicin; car – carboplatin; tra – trastuzumab; lap – lapatinib

**Table 2:** Cox’s Proportional Hazards Models – QuTILs and RNA Deconvolution Immune Phenotypes. RNA deconvolution estimates for multivariable model generation were selected by overlapping estimates across 40502 and 40603 in the top 15/bottom 15 by univariable Cox hazard ratio.

	CALGB 40502			CALGB 40G03		
Method						
	HR	95% CI	*p*	HR	95% CI	*p*
**Full Models**						
QuTILs Marginal % (decile)	**0.655**	**0.523-0.821**	**<0.000**	**0.998**	**0.996-0.999**	**0.012**
Resting Mast Cell (CBS)	1.090	0.953-1.245	0.209	1.033	0.961-1.111	0.379
Monoctye (QTS)	**1.481**	**1.186-1.850**	**<0.000**	1.059	0.991-1.131	0.089
Gamma Delta T Cell (CBS)	1.628	0.998-2.658	0.051	1.353	0.684-2.674	0.385
NK Cell (EPC)	0.934	0.359-2.428	0.888	0.448	0.180-1.118	0.085
Exhausted T Cell (ICAI)	0.591	0.270-1.297	0.190	0.886	0.604-1.299	0.536
Naïve CD4+ T Cell (ICAI)	0.928	0.492-1.751	0.817	0.696	0.341-1.420	0.316
**Filtered Models**						
QuTILs Marginal % (decile)	**0.664**	**0.533-0.829**	**<0.000**	**0.998**	**0.996-0.999**	**0.003**
Monocyte (QTS)	**1.425**	**1.136-1.787**	**0.002**	1.064	0.996-1.137	0.065

## Data Availability

The RNA sequencing data for CALGB 40603 analyzed in this study are publicly available in dbGaP, accession number phs001863.v1.p1 (https://www.ncbi.nlm.nih.gov/projects/gap/cgi-bin/study.cgi?study_id=phs001863.v1.p1). RNA sequencing data for CALGB 40502 and all digital pathology images are available to any investigator after scientific and regulatory review through Alliance Standardized Translational ‘Omics Resource (ASTOR) and Alliance Translational Research Program (TRP; https://www.allianceforclinicaltrialsinoncology.org/main/public/standard.xhtml?path=/Public/A-STOR).

## Abbreviations

bev: bevacizumab
pac: paclitaxel
nab: pac–nab–paclitaxel
ixa: ixabepilone
dox: doxorubicin
car: carboplatin
tra: trastuzumab
lap: lapatinib
